# Improving care for hypertension and diabetes in india by addition of clinical decision support system and task shifting in the national NCD program: I-TREC model of care

**DOI:** 10.1186/s12913-022-08025-y

**Published:** 2022-05-23

**Authors:** Devraj Jindal, Hanspria Sharma, Yashdeep Gupta, Vamadevan S. Ajay, Ambuj Roy, Rakshit Sharma, Mumtaj Ali, Prashant Jarhyan, Priti Gupta, Nikhil Srinivasapura Venkateshmurthy, Mohammed K. Ali, K M Venkat Narayan, Dorairaj Prabhakaran, Mary Beth Weber, Sailesh Mohan, Shivani A. Patel, Nikhil Tandon

**Affiliations:** 1 Centre for Chronic Disease Control, New Delhi, India; 2 Department of Endocrinology & Metabolism, All India Institute of Medical Sciences, New Delhi, India; 3 Faculty of Healthcare Management & Center for Excellence in Sustainable Development, Goa Institute of Management (GIM), Goa, India; 4 Department of Cardiology, All India Institute of Medical Sciences, New Delhi, India; 5 Public Health Foundation of India, Gurgaon, India; 6 Hubert Department of Global Health, Department of Family and Preventive Medicine, Emory University, Atlanta, GA USA; 7 Emory Global Diabetes Research Center, Hubert Department of Global Health, Emory University, Atlanta, GA USA; 8 Hubert Department of Global Health, Emory University, Atlana, GA USA

**Keywords:** Digital Health, Implementation science, Clinical Decision Support System, Task Shifting, Non-Communicable diseases, Scale up, Integration, Ayushman Bharat Initiative, NPCDCS, Care-coordinator

## Abstract

**Background:**

The growing burden of hypertension and diabetes is one of the major public health challenges being faced by the health system in India. Clinical Decision Support Systems (CDSS) that assist with tailoring evidence-based management approaches combined with task-shifting from more specialized to less specialized providers may together enhance the impact of a program. We sought to integrate a technology “CDSS” and a strategy “Task-shifting” within the Government of India’s (GoI) Non-Communicable Diseases (NCD) System under the Comprehensive Primary Health Care (CPHC) initiative to enhance the program’s impact to address the growing burden of hypertension and diabetes in India.

**Methods:**

We developed a model of care “I-TREC” entirely calibrated for implementation within the current health system across all facility types (Primary Health Centre, Community Health Centre, and District Hospital) in a block in Shaheed Bhagat Singh (SBS) Nagar district of Punjab, India. We undertook an academic-community partnership to incorporate the combination of a CDSS with task-shifting into the GoI CPHC-NCD system, a platform that assists healthcare providers to record patient information for routine NCD care. Academic partners developed clinical algorithms, a revised clinic workflow, and provider training modules with iterative collaboration and consultation with government and technology partners to incorporate CDSS within the existing system.

**Discussion:**

The CDSS-enabled GoI CPHC-NCD system provides evidence-based recommendations for hypertension and diabetes; threshold-based prompts to assure referral mechanism across health facilities; integrated patient database, and care coordination through workflow management and dashboard alerts. To enable efficient implementation, modifications were made in the patient workflow and the fulcrum of the use of technology shifted from physician to nurse.

**Conclusion:**

Designed to be applicable nationwide, the I-TREC model of care is being piloted in a block in the state of Punjab, India. Learnings from I-TREC will provide a roadmap to other public health experts to integrate and adapt their interventions at the national level.

**Trial registration:**

CTRI/2020/01/022723.

**Supplementary Information:**

The online version contains supplementary material available at 10.1186/s12913-022-08025-y.

## Background

The growing burden of hypertension and diabetes is one of the major public health challenges being faced by the health system in India [1, 2]. The Government of India (GoI) initiated the universal annual screening of hypertension and diabetes for all adults' aged 30 years and older as a component of comprehensive primary health care (CPHC) in 2017 to address the growing burden of Hypertension and Diabetes [3]. From the onset, GoI envisaged using technology to facilitate the screening and management of these conditions. First, Auxiliary Nurse Midwife (ANMs) were to use a mobile app for the identification of adults at high-risk for Non-Communicable Diseases (NCDs) like hypertension and diabetes at community level. Second, Medical Officers at Primary Health Centres (PHC) were to use a web-based system consisting of an electronic Case Record Form (eCRF) for screening high risk individuals for common chronic conditions and record patient information for routine management and referral. Collectively, this system is called the “GoI CPHC-NCD System.”

In addition to longitudinal health record keeping, digital technologies have been successfully used to improve the management of disease. Previous research has shown that the mHealth-based Clinical Decision Support System (CDSS) aided by task-shifting can facilitate guideline-based clinical management of patients and have a significant role in bridging these gaps in quality of care [4–6]. However, the development of technology is only a first step towards widespread adoption of such tools. The major barriers are the incorporation of technologies as an intrinsic component of the health system across the country and their acceptability by healthcare providers [7]. Many successful evidence informed public health interventions often fail to have an impact beyond the study period or the study population and are not widely adopted due to lack of systematic and efficient uptake by the relevant stakeholders, including governments and health systems in low- and middle- income country settings. This exacerbates the “know-do” gap. Through this study we are trying to understand (a) how best to integrate an evidence-based intervention (i.e. a CDSS) into a national public health program and (b) whether any modifications are required to incorporate the evidence-based intervention into the national program. The objective of this paper is to report the integration of an evidence informed digital health intervention with the national public health program for NCD and sharing the learnings that can be useful for other states in India and Low and Middle income countries in south-east Asia and beyond. In this paper, we describe in detail an academic-community-government partnership to incorporate a CDSS within the GoI NCD System and combine this with enhanced provider training and task-shifting strategies. The resulting model of care is known as I-TREC, or the Integrated- Tracking, Referral, Electronic decision support, and Care coordination platform. This experience provides lessons in the adaptations and modifications made in technologies and health care delivery systems to improve the quality of care for hypertension and diabetes.

## Methods

### History

Since 2010, the investigators from the I-TREC team (consisting of clinicians, public health specialists, health systems researchers, mHealth technical specialists) has been involved in incremental efforts for developing eCRF and CDSS for a range of conditions operational at varying levels of health care. The first CDSS was developed to improve diabetes care at tertiary care settings [8, 9], and this evolved into the development of CDSS for management of hypertension and diabetes at primary and secondary levels of health care [10–14]. In 2017, the state government of Tripura adopted one of the mHealth solutions for improving care for NCDs for the state-wide implementation [15]. However, most of this work has been implemented at a single level of the healthcare system (either at primary, or at secondary, or at tertiary care level). We sought to apply the lessons learned at single levels of the healthcare system to develop a “model of care” entirely calibrated for implementation within the current health system, spanning across all levels of care (Primary Health Centre-PHC, Community Health Centre-CHC, and District Hospital-DH), using the core principles of “task-shifting + technology” [16].

**Fig. 1:**
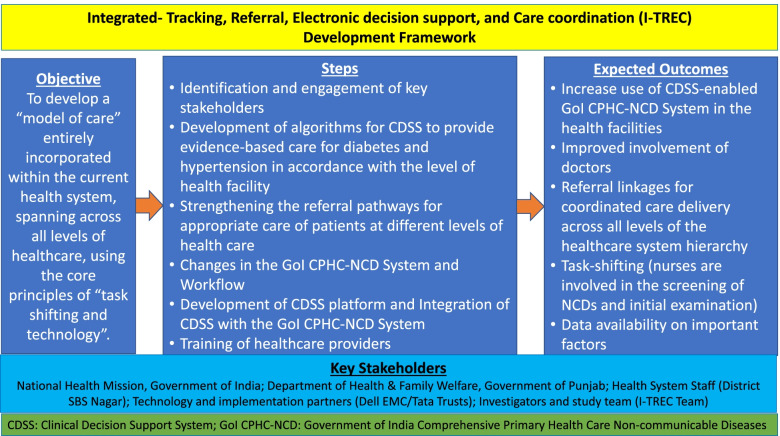
Integrated- Tracking, Referral, Electronic decision support, and Care coordination (I-TREC) Development Framework

The I-TREC team started the process of developing an integrated model of care for NCDs which can be implemented at national level after piloting in a block in Shaheed Bhagat Singh (SBS) Nagar district of Punjab by following these steps (Fig. 1):

#### Step 1: Identification and engagement of key stakeholders

We identified and engaged important key stakeholders, such as national health officials who were leading the implementation of GoI CPHC-NCD system, national and international public health experts working in the arena of NCDs and implementation and technology partners for the GoI CPHC-NCD system. Furthermore, we identified state and district health officials who were providing necessary support to the GoI for the implementation of the CPHC-NCD system and planning, implementing, and delivering NCD care in the state of Punjab. We signed an official Memorandum of Understanding with the state government of Punjab to implement and pilot test the I-TREC model in one administrative block of SBS Nagar. We created state, national and international advisory boards to identify the potential impact that can be achieved by the I-TREC and to help the I-TREC team in developing the initial roadmap to achieve the desirable goals. The members of different boards bring their vast experience in health policies, implementing national/international health programs related to NCD, and expertise in relevant clinical fields. We also created a Technology Working Group (TWG) of important stakeholders to identify and complete the necessary activities and actions to develop the CDSS-enabled GoI CPHC-NCD system (an organogram with role and responsibilities of different boards and groups is given in Additional file 1).

Along with meetings and interactions with different advisory boards, we visited the health facilities in Punjab and held meetings with local health care providers to assess the facility readiness and to finalize the framework required to develop new version of the GoI CPHC-NCD system. Through stakeholder engagement and based on previous research conducted by the investigators from the I-TREC team, literature review, and health facilities assessment, many challenges and needs were identified to successfully implement the I-TREC model of care. The main challenges identified were: low/limited acceptance among doctors for using technology; limited involvement of the non-physician healthcare workers in using the technology during the patient visits in the health facility; non-availability of hardware; and limited time for doctors to use the technology. The primary needs identified were ensuring that a patient goes to a higher facility or visits a specialist when it is required and using the technology to support the healthcare providers in providing better care to the patients with diabetes and hypertension. The TWG had multiple meetings (in-person and virtual) to undertake and complete the activities required for the development of the CDSS-enabled NCD system.

#### Step 2: Development of algorithms for CDSS to provide evidence-based care for diabetes and hypertension in accordance with the level of health facility

Experts from cardiology, endocrinology, public health, and digital health reviewed national and international guidelines to develop guideline-based clinical algorithms which can be easily implemented by the healthcare providers in their routine clinical practice within the constraints of government-run health facilities in rural India [17–21]. Experts developed the algorithms for the management of hypertension and diabetes which can be implemented at all levels of care (PHC, CHC, and DH). While generating recommendations, algorithms also consider presence of co-morbidities (such as heart disease, chronic kidney disease, asthma, etc.); current medication; lab investigations, anthropometric, and clinical parameters. Based on the available information, CDSS algorithms can suggest change in medication, adding a new medication and up-titration or down-titration.

#### Step 3: Strengthening the referral pathways for appropriate care of patients at different levels of health care

The hierarchy of the health system in India has created the referral pathways for appropriate care of patients, as patients might need to refer to a higher level of care and from there to a health facility near to them. Through expert consultations and discussions with the TWG, referral linkages between different levels of care were strengthened by in-built referral thresholds for healthcare providers, notifications to the referred facilities, and by ensuring availability of the health record of the referred patients at different levels of care. Logics for up-referral from the PHC to CHC/DH or CHC to DH and down-referral from higher levels of health facilities to lower levels, where maintenance care could be managed were built. One example of such referral scenario is given in Additional file 2.

#### Step 4: Changes in the National NCD System and Workflow

**Fig. 2:**
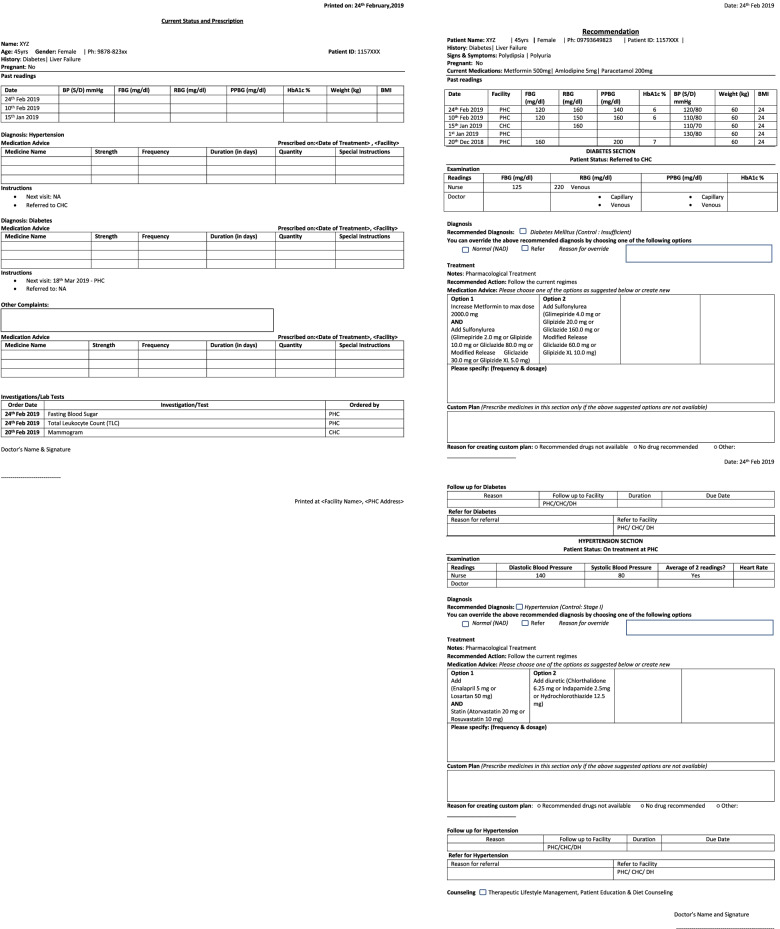
Screenshots of Printouts of Prescription and CDSS recommendations

The CDSS integration with the National NCD system required significant efforts and changes, as algorithms and referral linkages were designed to provide support across all levels of care. To make sure both the systems communicate with each other, the TWG mapped each variable. The group listed out the variables which were part of the current version of the system and variables required for CDSS to provide evidence-based recommendations. Few variables were added in the eCRF to capture the information required to inform the CDSS and compute the management plan. Changes were made in the user interface of the eCRF to receive the recommendations from the CDSS. A new format for capturing drug history along with additional details about the drug section was implemented to provide the necessary information. The workflow within the system was redesigned to make it user friendly and conducive for the in-putting of all variables required for the CDSS by the non-physician health care provider (nurses posted at government health facilities). The print-out feature was provided in the CDSS-enabled version of the GoI CPHC-NCD system. Two types of printouts (Fig. 2) were suggested and incorporated in the GoI CPHC-NCD NCD system; one for a prescription that can be given to the patient and other for sharing the CDSS recommendations with the doctor.

#### Step 5: Development of CDSS platform and Integration of CDSS with the GoI CPHC-NCD NCD System

**Fig. 3:**
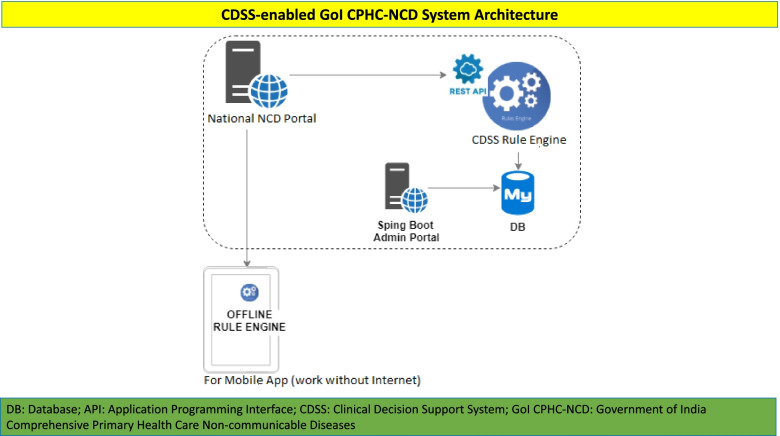
CDSS-enabled GoI CPHC-NCD System Architecture

The CDSS platform was designed in such a way that it should be capable of integration with the online CPHC-NCD system and be able to push the rule-engine in the offline mode if there is any requirement. The overall architecture of the CDSS-enabled GoI CPHC-NCD system was designed in such a way that Application Programming Interface of web based national NCD system (eCRF) and CDSS platform can interact with each other (Fig. 3).

For the integration of CDSS with eCRF, a detailed plan was prepared and executed. The CDSS-enabled GoI CPHC-NCD system was deployed by following these three steps:

o The GoI CPHC-NCD system was being used across states in government health facilities for Population based screening under the Ayushman Bharat initiative. Beneath the hood the NCD system was modified to incorporate the CDSS engine and necessary changes were made to the associated workflow to create the eCRF for the I-TREC model of care.
p Following the development of the CDSS-enabled system, the solution was deployed in a staging/testing instance to ensure a thorough validation before it was rolled in the healthcare facilities in Punjab. The TWG tested and validated the data flow, integration, CDSS recommendations, and the user experience. The initial testing was done by the software developers to ensure the platform does not have any critical errors. By using Postman (an interactive and automatic tool for verifying Application Programming Interface), the TWG team conducted a thorough testing of the clinical case scenarios to ensure CDSS provides correct recommendations. Also, the TWG tested CDSS-enabled GoI CPHC-NCD system at the Out-Patient Department of endocrinology and cardiology of All India Institute of Medical Sciences (AIIMS), New Delhi on thirty-two patients to check the operational feasibility. The discrepancies found during testing were recorded in an excel file against each case and were reported to the software team for correction. In addition, the admin system has been built for the CDSS platform, which can be used by designated users to make changes in the rule-engine, when required.
q After incorporating necessary feedback, the CDSS-enabled GoI CPHC-NCD system was then made available to the healthcare providers in Punjab.

#### Step 6: Training and handholding of healthcare providers

A three-day training program was conducted for the healthcare providers (25 medical officers and 27 nurses) in the district SBS Nagar. We used the training material and agenda developed by the implementation partner for “training of trainers” at national level and made relevant modifications in the training material to include information about the CDSS-enabled GoI CPHC-NCD system**.** The training program was divided into two categories: 1) refresher training on the medical content; and 2) training and orientation of the CDSS-enabled GoI CPHC-NCD system. On Day 1 of training of the medical officers, the training course focus was the recent updates on the management of hypertension and diabetes, screening of cancers, and discussion of case studies. On Day 2, the ITREC team presented a comprehensive demonstration of the CDSS-enabled GoI CPHC-NCD system and explained the modified workflow. On Day 3, medical officers did hands-on practice with the system. Similarly, on Day 1 of the training sessions for the nurses, an overview of hypertension, diabetes, and cancers was given. On Day 2, a detailed demonstration of the CDSS-enabled GoI CPHC-NCD system was given. Nurses were informed and trained about their primary responsibilities while using the GoI CPHC-NCD system. A nurse in the health facility is supposed to fill the registration section (if patient is not already registered) and Initial assessment section (which includes entering information about vitals, medical history, and drug history). Along with the initial assessment, nurses can assist the doctors in taking final print-out of the prescription and delivering lifestyle advice. On Day 3, along with the hands-on practice of the system, a refresher training of anthropometry was given to the nurses. The training sessions of the medical officers and nurses on Days 2 and 3 were conducted together, so they can understand each other’s roles and responsibilities within the CDSS-enabled GoI CPHC-NCD system*.* After the training program, orientation of the additional staff at healthcare facilities along with the handholding of the healthcare providers was done by the I-TREC team. The training, orientation, and handholding was done to promote awareness about the I-TREC model of care and ensure buy-in.

## Results

The above activities, constant efforts from technical working group, and inputs from key stakeholders led to the development of a model of care “I-TREC” based on two core principles: task shifting and technology.

### Task shifting

Modifications were made in the GoI CPHC-NCD system and workflow for enabling the efficient implementation of the I-TREC model. The fulcrum of care has been shifted from the doctors to the nurse/non-physician healthcare provider. In the previous version of the GoI CPHC-NCD system (before the integration of the I-TREC model of care), a doctor was supposed to enter a lot of details from the patient's visit (medical history, drug history, etc.) as part of the initial assessment. However, in the new integrated version, a non-physician healthcare worker/nurse has access/permission to enter the details of the initial assessment (which can be vetted by a doctor), and the doctor spends his/her time focusing on the examination section which includes a brief patient profile, key medical and drug history findings, CDSS recommendations, and final prescription. So, in the modified clinic workflow, the nurse first interacts with the patient, does the initial assessment by using the GoI CPHC-NCD system, following which the in-built CDSS works at the backend and generates guideline-based recommendations for the doctors. Doctors thereby receive an appropriately triaged patient with evidence-based recommendations, which ensures efficient utilisation of their time and enables objective decision making. To ensure, the nurse can perform the tasks appropriately a refresher training program was prepared.

### Technology

**Fig. 4:**
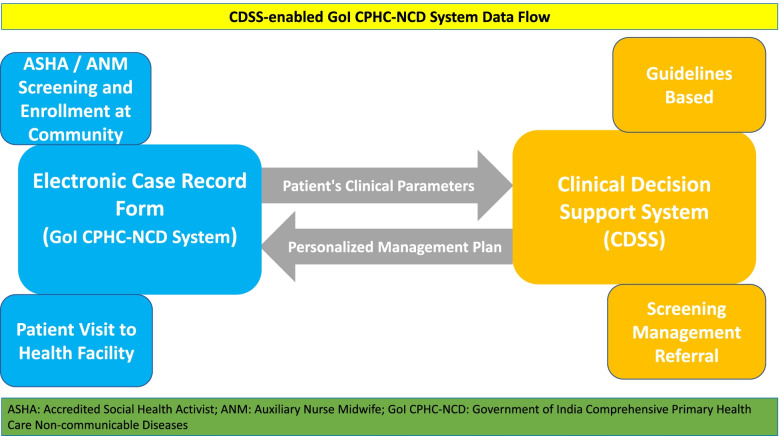
CDSS-enabled GoI CPHC-NCD System Data Flow

The CDSS-enabled GoI CPHC-NCD system has two principal components: electronic case record form (eCRF) and clinical decision support system. Within the CDSS-enabled GoI CPHC-NCD system, eCRF and the CDSS system communicate with each other and exchange data. Healthcare providers enter the data of patients in the eCRF through the CPHC-NCD system or in the community using the ANM NCD App. Anonymized data are sent by the eCRF to the CDSS platform, which computes and sends back the recommendations/management plan based on the evidence-based guidelines to the eCRF (Fig. 4). The CDSS engine does not store any patient specific data, or any other information related to patient visit. It only derives information from the GoI CPHC-NCD system to generate management prompts which are then delivered to the doctors/ designated health care worker via the system. The CDSS-enabled GoI CPHC-NCD system has features that supports all the three essential steps (screening, management, and follow-up) of chronic disease management. The CDSS platform considers many parameters to provide recommendations such as clinical information, medical history, drug history, personal history, level of health facility, availability of drugs, and investigations. For example, for patients with diabetes, the CDSS platform provides recommendations based on the availability of lab test values such as glycated haemoglobin (HbA1C) & fasting blood glucose (FBG) & post-prandial blood glucose (PPBG); or HbA1C & FBG; or FBG & PPBG; or only FBG; or only random blood glucose. Along with the oral-anti-hyperglycaemic agents and anti-hypertensive medications, the CDSS platform also supports healthcare providers in initiating and iterating of drugs such as insulin, statins, and aspirin. In addition, the GoI CPHC-NCD system gives impetus to lifestyle management and displays the section on lifestyle as a priority. Doctors are prompted to assess lifestyle behaviours and review the lifestyle recommendations with patients. The CDSS-enabled GoI CPHC-NCD system has in-built prompts to assure appropriate referral mechanisms across health facilities based on the clinical parameters of patients. The system enhances care coordination through workflow management and dashboard alerts. Furthermore, it ensures seamless availability of the integrated patient database across all levels of health facilities and across longitudinal patient visits to enable healthcare providers to take an informed decision. The GoI CPHC-NCD system has many security features to ensure confidentiality. Information access is restricted as per the roles and Electronic Health Record (EHR) standard security best practices are also implemented. It is hosted in the Government of India data centre and Ministry of Electronics and Information Technology (MeitY) data centre best practices and guidelines are followed. The CDSS engine is also hosted in the same environment and uses anonymized patient information to do the calculations. The patient clinical data is linked with the system-generated unique health identification number and the health facility (PHC or CHC or DH) while being sent to the CDSS engine. Both the systems (CPHC-NCD System and CDSS engine) communicate with each other in real-time through open API integration.

### Workflow

**Fig. 5:**
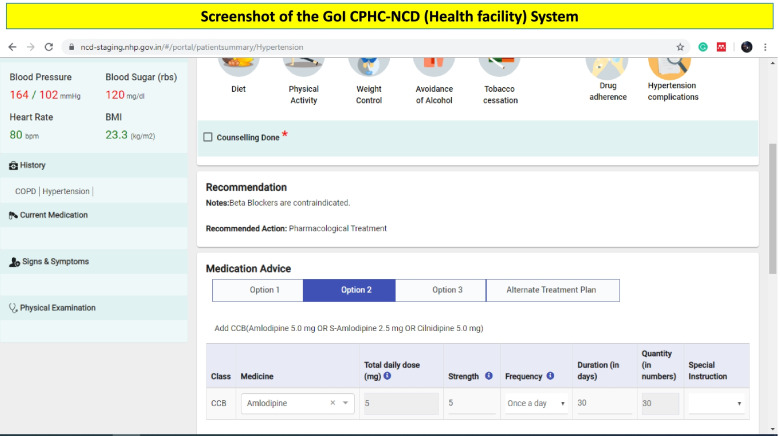
Screenshot of the GoI CPHC-NCD (Health facility) System

In the new version of the GoI CPHC-NCD system, a patient visiting the health facility, first interacts with the nurse. Once the nurse has completed the initial assessment, the patient visits the doctor, who can further view the assessment done by the nurse on his/her system. Doctors are free to make changes and order any tests at any time during the patient visits. Once the doctor completes the examination section, the CDSS works in the backend and provides evidence-based recommendations (Fig. 5). The CDSS supports the physician in providing an accurate diagnosis of hypertension/diabetes and in prescribing an evidence-based management plan. This includes the name of the generic drug with dosage and follow-up time based on the patient’s clinical parameters. It also provides information on contraindications for drugs. The final prescription can be printed by either a doctor or nurse by using the system and can be given to the patient after the approval of the doctor. The doctors at the health facility can disagree with the recommendations provided by the CDSS and provide their reason for disagreement. In health facilities, where the doctor is unable to use technology because of a lack of hardware, space, internet, or frequent changes in the rosters, the new version of the system has a print-out option for the CDSS recommendations. This print-out provides information about the brief profile of the patient, clinical parameters of the last five visits, and recommendations on diagnosis and management plan.

Table 1 describes the important components of I-TREC model of care designed to address the existing challenges and needs of the healthcare delivery system.

**Table 1:** Health system needs and challenges and corresponding Components/Solutions of the I-TREC Model of Care

Health System Needs and Challenges	“I-TREC model of care” Components	Adaptations made for integration with GoI CPHC-NCD System
Supporting the healthcare providers in providing better care to the patients	Clinical Decision Support System (CDSS)	The CDSS for hypertension and diabetes has been integrated with GoI CPHC-NCD system to support the doctors in providing evidence-based care to patients at all the three essential steps of chronic disease management which are: screening, management, and follow-up. *^a^,^b^,^c^,^d^
Limited acceptance by doctors in using technology	Task-shifting to ensure the doctor spends time on the important tasks	The fulcrum of care (initial assessment of the patient during the health facility visit) has been shifted from the doctors to the nurse/non-physician healthcare provider. In the new workflow, a non-physician healthcare provider/nurse can do some of the tasks like using the technology solution to enter the details of the patients such as registration (if required), medical history, drug history, and measurements. *^a^,^b^,^d^
Patient referral	Strengthening the referral pathways	The CDSS-enabled GoI CPHC-NCD system has in-built referral thresholds based on the clinical parameters of the patients and level of health facility. It ensures the availability of the health record at different levels of care and sends the notifications of patient’s referral to the referred facilities. *^a^
Limited involvement of the non-physician healthcare workers in using the technology	Changes in the technology/GoI CPHC-NCD system for the better involvement of the non-physician healthcare workers	In the new version “CDSS-enabled GoI CPHC-NCD System”, the non-physician healthcare worker/nurse has got the access/permission to enter the details of the initial assessment (which can be vetted by a doctor). *^b^,^c^
Non-availability of hardware or limited time for doctors to use the technology	Addition of print-out functionality	In the CDSS-enabled GoI CPHC-NCD system, print-out functionality has been added. Through this functionality, a non-physician healthcare worker/nurse can take a print-out of the patient brief profile (which includes important findings of the current visit and previous visits) and CDSS recommendations. If a doctor is not using any computer/laptop/tablet because of limited resources or time constraint, he/she can review the profile and CDSS recommendations on the print-out. *^b^,^c^,^d^

Source of suggestions for these changes/additions

^a^Key stakeholders’ suggestions

^b^Based on previous research conducted by the investigators from the I-TREC team (reference # 8–16)

^c^Health facility assessment

^d^Literature

## Discussion

This paper describes the systematic development of a model of care “I-TREC” to deliver evidence-based care for hypertension and diabetes in government health facilities. During meetings and discussions with stakeholders, one of the critical barriers reported was a lack of enthusiasm among healthcare providers (especially doctors) in using the GoI CPHC-NCD system. Previous studies have also reported that it is difficult for doctors to spend time on any technological solution in government health facilities due to lack of resources and time constraints [11, 15]. To overcome such barriers, the principle of task-shifting was implemented, allocating information deemed extraneous to a physician to a non-physician health care provider such as a nurse. The key stakeholders envisaged the expected outcomes from the implementation of the CDSS-enabled GoI CPHC-NCD system, which are: increased use of the GoI CPHC-NCD system (CDSS-enabled) in the health facilities and improved involvement of doctors in using the CPHC-NCD system because of task-shifting; improved continuum of care because of in-built referral linkages for coordinated care delivery across all levels of the healthcare system hierarchy; and data availability on important factors such as non-availability of the drugs or doctor’s opinion of rejecting CDSS recommendations.

The CDSS-enabled GoI CPHC-NCD NCD system has some limitations. The CPHC-NCD system is unable to work without internet reception. During the development of the CDSS-enabled GoI CPHC-NCD system, two approaches were considered to provide offline functionality. One approach was to develop an android app for the health facilities which can be used by the nurse or doctor with or without the internet. The second approach was to run the NCD server locally in a desktop/computer and link the users of that health facility with the local NCD server. The first approach of using the android app was not considered further because of issues related to the storage and security of the offline data in the device. The technical working group worked on the second approach and developed the offline functionality by creating a local NCD server. However, this approach was also not found to be feasible while doing the internal testing as it required technical support every day at the health facility and the large size of the offline-server file made it practically difficult to download and transfer to the different health facility. Another limitation of the CDSS-enabled GoI CPHC-NCD system is that the CDSS only provides recommendations for hypertension and diabetes only but the eCRF can also store information about the screening of three types of cancers (breast, cervical, and oral). In due course, the CDSS-enabled GoI CPHC-NCD system can be extended to include CDSS for cancer screening. Other limitations include dependencies of any technological solution such as the availability of manpower and infrastructures.

## Conclusion

To our knowledge, this is the first study that describes the detailed process of strengthening the GoI CPHC-NCD system by integrating CDSS platform and task shifting to improve care for hypertension and diabetes in India. Adaptations were implemented in the NCD system in our single pilot site in Punjab. Further integration with the nationwide system will involve multiple stakeholders. Learnings from the I-TREC project will assist in further refinement and fine-tuning of the CDSS-enabled GoI CPHC-NCD system for meeting the requirement of the Indian health system to deliver quality NCD care to people of India. The effectiveness and limitations of the CDSS-enabled GoI CPHC-NCD system are currently being evaluated through “I-TREC” project in Punjab, India (trial registration number CTRI/2020/01/022723).

## Supplementary Information

**Additional file 1.**

**Additional file 2.**

## Data Availability

Data sharing is not applicable to this article as no datasets were generated or analysed during the current study.

## Abbreviations

AIIMS: All India Institute of Medical Sciences
ANM: Auxiliary Nurse Midwife
CDSS: Clinical Decision Support Systems
CHC: Community Health Centre
CPHC: Comprehensive Primary Health Care
DH: District Hospital
eCRF: Electronic Case Record Form
FBG: Fasting blood glucose
GoI: Government of India
HbA1c: Glycated haemoglobin
ITREC: Integrated- Tracking, Referral, Electronic decision support, and Care coordination
NCD: Non-Communicable Diseases
PHC: Primary Health Centres
PPBG: Post-prandial blood glucose
SBS: Shaheed Bhagat Singh
TWG: Technology Working Group
