# Supplementary material for: Improving care for hypertension and diabetes in india by addition of clinical decision support system and task shifting in the national NCD program: I-TREC model of care
Source: BMC Health Serv Res. 2022 May 23;22:688. doi: 10.1186/s12913-022-08025-y (PMC9125907; doi:10.1186/s12913-022-08025-y)
Supplement: Supplementary file 1 — Additional file 1. [file 12913_2022_8025_MOESM1_ESM.docx]

| **Executive Committee** |
| --- |

**State Advisory Committee**

**Appendix 1**

**ORGNAOGRAM: I-TREC MODEL OF CARE**

**Steering Committee**

**National Advisory Committee**

**International Advisory Committee**

| **Operation Group Committee** |
| --- |

| **Technology Working Group** |
| --- |

| **Steering Committee** | | |
| --- | --- | --- |
| **Structure** | **Principal Investigators and Co-Investigators**  Nikhil Tandon  Sailesh Mohan  D Prabhakaran  Shivani Patel  Mary Beth Weber  Mohammed K Ali  KM Venkat Narayan | **External**  NHLBI representative  Government of Punjab representative |
| Roles and responsibilities | - To monitor progress and achievement of milestones and to further communicate findings with external agencies and partners. - The Steering Committee will comprise the Investigators, a representative of the funding institution, and a representative of the government partner - This committee will convene in person on an ad hoc basis - Convenes quarterly | |

| **Executive Committee** | | |
| --- | --- | --- |
| **Structure** | Nikhil Tandon (PI)  Sailesh Mohan  D Prabhakaran  Ambuj Roy  Yashdeep Gupta | Shivani Patel  Mary Beth Weber  Mohammed K Ali  KM Venkat Narayan |
| Roles and responsibilities | - To drive the scientific goals of the study - Convenes quarterly | |

| **Operations Group Committee** | | |
| --- | --- | --- |
| **Structure** | **Principal Investigators and Team Members**  Nikhil Tandon  Sailesh Mohan  D Prabhakaran  Shivani Patel  Mary Beth Weber  Hanspria Sharma  Rakshit Sharma  Devraj Jindal  Prashant Jarhyan  Nikhil SV  Mumtaj Ali  Yashdeep Gupta  Ambuj Roy  Priti Gupta | **External**  NHLBI representative |
| Roles and responsibilities | - Responsible for executing activities related to I-TREC goals. - Convenes weekly through teleconferencing to keep all members of the Operations Committee informed of important activities and developments to ensure a coordinated effort on the ground. | |

| **National and International Advisory Committee** | | | |
| --- | --- | --- | --- |
| **Structure** | **State Advisory Committee**   - Chair- Additional Secretary Health & Family Welfare, Punjab - Members - Convenor- Dr Nikhil Tandon - Mission Director, National Health Mission - Director Health & Family Welfare, Punjab - State Programme officer, NPCDCS - Dr Rajesh Kumar, Professor & Head, School of Public Health, PGI, Chandigarh - Civil Surgeon, Shaheed Bhagat Singh Nagar - SMO District Hospital, Shaheed Bhagat Singh Nagar - SMO Community Health Centre, Mukandpur, , Shaheed Bhagat Singh Nagar - Special Invitee- Professor & Head, Department of Medicine, Government Medical College and Hospital, Amritsar - Special Invitee- Professor & Head, Department of Medicine, Guru Gobind Singh Medical College and Hospital, Faridkot - Special Invitee- Professor & Head, Department of Medicine, Government Medical College and Hospital, Patiala | **National Advisory Committee**   - Chair- Dr Vinod Paul, Member, NITI Aayog [National Institution for Transforming India] - Members: - Manoj Jhalani, Additional Secretary and Managing Director, National Health Mission, Ministry of Health and Family Welfare, Government of India - Rajeev Kumar, Director (NCD) Ministry of Health and Family Welfare, Government of India - Principal Secretary (Ex-officio), Health and Family Welfare, Government of Punjab - Rajani Ved, Executive Director, National Health Systems Resource Centre - Henk Bekedam, WHO, SEARO - Convenor - Nikhil Tandon | **International Advisory Committee**   - Chair- Anushka Patel, George Institute for Global Health, Sydney - Members:   - Kamlesh Khunti, Professor, Primary Care Diabetes, Leicester   - Lara Fairall, Associate Professor, Knowledge Translation Unit, University of Cape Town, South Africa   - Pablo Perel, Associated Professor, London School of Hygiene and Tropical Medicine   - Anil Kapur, Chairman, Board of Directors, World Diabetes Foundation   - Neil Poulter (President of the International Society of Hypertension)   - Convenor - Nikhil Tandon |
| Roles and responsibilities | - To provide inputs into developing a scalable and generalizable model of coordinated healthcare delivery for hypertension and diabetes - Formulating best practices for technical capacity building within public healthcare systems to ensure program sustainability, - Indicators of quality of program implementation (structure, process, outcomes) to assess the improvement in quality of care for hypertension and diabetes detection and management in India and similar settings - Convenes annually for National Advisory Board members and virtually for International Advisory Board | | |

| **Technical Working Group** | | | | | |
| --- | --- | --- | --- | --- | --- |
| **Structure** | **AIIMS**  Prof. Nikhil Tandon  Prof. Ambuj Roy  Prof. Yashdeep Gupta  Dr. Hanspria Sharma  Mr. Rakshit Sharma | **CCDC**  Prof. D Prabhakaran  Dr. Ajay VS  Dr. Dev Raj Jindal | **Emory**  Dr. Shivani Patel | **Dell EMC**  Ms. Sunita Nadhamuni  Mr. Mallari Kulkarni  Ms. Supriya Prabhakar  Ms. Sruti Sridhar  Mr. Kiran Kumar Erugu  Mr. Eshan Nanda  Mr. Irfan Ulla Syed | **Tata Trusts**  Dr. Aman Singh  Dr. Prashant Pathak |
| **Roles & responsibilities** | - Execute the activities necessary to develop the CDSS-enabled National NCD System - Developing architecture for the integration of CDSS with National NCD System - Making changes in the software and advocating new patient workflow to implement the CDSS-enabled National NCD System in Punjab - Informing the progress of activities to the important stakeholders - Convenes as required | | | | |
