# Supplementary material for: Improving care for hypertension and diabetes in india by addition of clinical decision support system and task shifting in the national NCD program: I-TREC model of care
Source: BMC Health Serv Res. 2022 May 23;22:688. doi: 10.1186/s12913-022-08025-y (PMC9125907; doi:10.1186/s12913-022-08025-y)
Supplement: Supplementary file 2 — Additional file 2. [file 12913_2022_8025_MOESM2_ESM.docx]

| Referral Scenario – Data Flow | |
| --- | --- |
| Referral Scenario | Expected Result at National NCD System of the referred CHC |
| Patient referred by the doctor form PHC to CHC for the management of Diabetes. | 1. After successful log in to the CHC system, the individual details should appear in the landing page of the CHC System.  2. In the status column for Diabetes it should appear as 'Referred by PHC'.  3. On click in the individual details, the page should land on the Initial assessment Vital page.  4. In the Vital page, the PHC updated data should display.  5. In the left panel, the latest PHC updated data should display.  6. If Nurse/Doctor updated any initial assessment details in CHC then the details should Save successfully.  7. The updated initial assessment details in CHC should appear in the Left panel.  8. Upon click on Diabetes it should land in Examination page with updated examination details.  9. Save & Continue in the Examination page, it should land on the Diagnosis page.  10. In the diagnosis page, the Recommended action should display the value what CDSS platform has provided.  11. In Diagnosis page, by selecting non-recommended action, doctor can override the CDSS recommendation.  12.Upon click on Continue button (agree with CDSS Diagnosis) in the diagnosis page, it should land on the Treatment page.  13. In the Treatment page Recommendation (notes and recommended action) / Medication Advice / Follow up should display the result as per what CDSS has returned.  14. Upon click on Save button in the treatment page all the details should save successfully.  15. After successful save it should return to the Examination page and Status should change from 'Examined' to 'On Treatment'. |

Appendix 2
